# Towards a Multi-Informant Assessment of Math Anxiety in Primary School Children

**DOI:** 10.3390/bs16101838

**Published:** 2026-10-08

**Authors:** Laura Di Leonardo, Maria Anna Donati, Caterina Primi

**Affiliations:** Department of Neuroscience, Psychology, Drug Research and Child Health (NEUROFARBA), Section of Psychology, University of Florence, 50135 Florence, Italy; laura.dileonardo@unifi.it (L.D.L.); mariaanna.donati@unifi.it (M.A.D.)

**Keywords:** math anxiety, multi-informant assessment, ES-AMAS, primary school children, mathematics education

## Abstract

A comprehensive understanding of children’s psychological functioning benefits from a multi-informant approach, gathering information from parents, teachers, and, when possible, the children themselves. This strategy enhances reliability, validity, and contextual coverage, addressing the limitations of relying solely on self-reports in young populations. Building on this rationale, the present study aimed to develop and validate parent- and teacher-proxy versions of the Abbreviated Math Anxiety Scale for elementary school children, complementing child self-report measures. A total of 1146 children (50.66% females) aged 6 to 11 years, along with their parents and teachers, participated in the study. Confirmatory factor analyses supported the hypothesized two-factor structure, reflecting learning-related and evaluation-related math anxiety. All scales demonstrated good internal consistency, and correlations among informants were small-to-medium, highlighting the unique contribution of each perspective. Multi-informant scores were negatively associated with children’s mathematical competence, and each informant’s perspective uniquely explains variance in mathematical competence, with teachers’ ratings showing the strongest association. These findings underscore the importance of a multi-informant assessment for capturing children’s math anxiety more accurately, enabling a nuanced understanding of its impact on academic outcomes and supporting early identification and targeted interventions.

## 1. Introduction

A comprehensive understanding of a child’s psychological functioning is best achieved through a multi-informant approach, which gathers information from key figures in the child’s life, typically parents and teachers (De Los Reyes et al., 2015). This method is widely employed in the psychological assessment of primary school children’s internal affective states, such as anxiety and depression, and has demonstrated high reliability and validity in clinical contexts. A well-known example of a multi-informant tool is the Child Behavior Checklist (CBCL; Achenbach & Rescorla, 2001), which gathers parallel reports from parents and teachers to assess both externalizing and internalizing problems. Extensive literature supports the use of multiple informants when assessing children’s functioning (e.g., Achenbach, 2006; Hunsley & Mash, 2007; Kendall & Flannery-Schroeder, 1998). Children, particularly the younger ones, often lack the cognitive and linguistic skills required to fully comprehend questionnaire items (Borgers et al., 2003; Varni et al., 2007) or to use Likert-type scales effectively (Mellor & Moore, 2014). Furthermore, they may struggle to recognize or express their emotional states (Pons et al., 2004). Consequently, relying solely on self-reports can limit the accuracy of assessments (De Los Reyes et al., 2015). Incorporating data from both parents and teachers offers several advantages, including broader coverage of contexts and a more accurate representation of the child’s emotions and behaviors.

From a psychometric perspective, integrating multiple sources of information enhances both reliability and validity of psychological assessments (Renk & Phares, 2004). This method acknowledges the complex and multifaceted nature of psychological constructs, yielding a more detailed and accurate depiction of children’s emotional and behavioral states, and ultimately leading to more effective support and intervention strategies. This approach also enhances the incremental validity of measurement tools, as each informant contributes unique insights that together provide a more nuanced understanding of the child’s functioning. Research has shown significant informant effects in explaining children’s functioning, underscoring the added value of combining adult and child perspectives (e.g., Izquierdo-Sotorrío et al., 2016).

In summary, a multi-informant approach to the psychological assessment of children offers several clear advantages: Greater comprehensiveness, improved reliability, and the capacity to capture contextual variability, thereby supporting more accurate evaluations and better-targeted interventions. Building on this rationale, the present study aimed to enhance the assessment of math anxiety in children, a domain in which self-report measures are predominantly used despite their reduced reliability in younger populations (Edelbrock et al., 1986). Alternative strategies, such as multi-informant assessment, are therefore essential for integrating multiple perspectives and enhancing the accuracy and reliability of math anxiety measurement in early childhood.

Math anxiety refers to the feeling of tension, apprehension, or fear experienced when engaging in math-related activities (Ashcraft & Ridley, 2005). It can emerge as early as age six (Aarnos & Perkkila, 2012) and can significantly affect math achievement (Barroso et al., 2021; Galla & Wood, 2012; Ramirez et al., 2016), academic learning (Krinzinger et al., 2009; Wu et al., 2012), and overall well-being (Krinzinger et al., 2009; Ramirez et al., 2013). Evidence from primary school students highlights the importance of understanding math anxiety to design effective educational strategies and targeted interventions. Reliable assessment tools are essential for identifying at-risk children and tracking changes over time, thereby enabling the evaluation of interventions (Balt et al., 2022). Early detection is particularly valuable, as timely interventions can simultaneously enhance mathematical competence and reduce subject-specific anxiety (Balt et al., 2022; Petronzi et al., 2019). Consequently, establishing reliable and valid methods for measuring math anxiety in children is a critical priority.

Several measures have been developed to assess math anxiety in children (Barroso et al., 2021). Some were specifically designed for primary school populations (Jameson, 2013; Petronzi et al., 2019; Wood et al., 2012), whereas others were adapted from existing scales (Chiu & Henry, 1990; Suinn et al., 1988; Wigfield & Meece, 1988). These scales vary in target population and length, yet many share methodological limitations, including small sample sizes, limited validity evidence, lack of test–retest analyses, insufficient confirmatory procedures for scale dimensionality, and absence of normative data (Eden et al., 2013; Harari et al., 2013). Importantly, to our knowledge, no measure has been specifically developed to provide a multi-informant assessment of children’s math anxiety. Thus, there is a need not only for psychometrically sound measures of children’s math anxiety, but also for instruments that can capture this construct from the perspectives of different informants.

Among the existing measures, the Abbreviated Math Anxiety Scale (AMAS; Hopko et al., 2003) represents a particularly promising starting point for such an approach. The AMAS (Hopko et al., 2003) is among the most extensively validated measures of math anxiety. It has been adapted into several languages (e.g., Cipora et al., 2015; Primi et al., 2014; Vahedi & Farrokhi, 2011) and, more recently, for use with children (Carey et al., 2017; Caviola et al., 2017; Li et al., 2022; Martín-Puga et al., 2022; Megreya et al., 2023; Milovanović & Branovački, 2021; Primi et al., 2020; Szczygieł, 2019).

To date, specific adaptations of the AMAS have been developed for children aged 6–7 (Primi et al., 2020) and 8–11 (Caviola et al., 2017). Consistent with the original version (Hopko et al., 2003), these adaptations assess two dimensions of math anxiety: Learning-related math anxiety, i.e., *“When another student solves a sum on the board”/“Watching another student solve a math problem”*, and Evaluation-related math anxiety, i.e., *“When you are taking a math test at school”/“Doing a written math test”*. The elementary school AMAS, consisting of nine items, is brief, easy to administer, and can be completed quickly by young children, making it well-suited for large-scale assessments and repeated monitoring. Its multidimensional structure allows for a more comprehensive evaluation of the anxiety experience than unidimensional measures, while its simple wording facilitates comprehension and reduces the risk of misinterpretation. In addition to its practicality, the instrument has shown strong psychometric properties (Caviola et al., 2017; Primi et al., 2020), supporting its reliability and validity. Unlike many other math anxiety scales, which have been validated in limited settings, it has also been tested for cultural-linguistic invariance and has been shown to be invariant across its Italian and English versions (Primi et al., 2020).

Building on this evidence, the present study aimed to address this gap by developing a multi-informant measure of children’s math anxiety based on the AMAS (Hopko et al., 2003), extending its use beyond child self-report to incorporate reports from multiple informants. Specifically, it aimed to develop parent- and teacher-proxy versions of the elementary school AMAS (Caviola et al., 2017; Primi et al., 2020) adapted for administration to parents (Elementary School-Abbreviated Math Anxiety Scale-Parent [ES-AMAS-P]) and teachers (Elementary School-Abbreviated Math Anxiety Scale-Teacher [ES-AMAS-T]). The study also sought to investigate their psychometric properties to determine whether these versions represent reliable and valid tools for assessing math anxiety in children. The decision to involve parents and teachers as informants was grounded in their complementary opportunity to observe the child across different contexts. Teachers can observe children during school-based mathematics activities (e.g., math lessons and assessments) and can therefore provide insights into how math anxiety manifests in the classroom. Parents, in contrast, can offer a complementary perspective based on their knowledge of the child’s emotional and behavioral responses to mathematics-related activities, particularly in the home context (e.g., doing homework or studying mathematical topics). Importantly, the parent version was designed to capture parents’ perceptions of how their child experiences and responds to mathematics-related situations, rather than requiring parents to report only on behaviors they have directly observed. This is particularly relevant for situations addressed by the AMAS that typically occur at school and, therefore, are not directly accessible to parents. Parents’ perceptions of their child’s experiences in those situations may be informed by behaviors observed during mathematics-related activities at home, as well as by what children communicate about their experiences with mathematics. In this way, parents’ reports provide a distinct source of information that complements both children’s self-reports and teachers’ observations.

Accordingly, we first assessed whether both proxy versions retained the two-factor structure originally identified by Hopko et al. (2003) and subsequently validated in child self-report versions (Caviola et al., 2017; Primi et al., 2020), and we examined their reliability in terms of internal consistency. Based on previous studies (Caviola et al., 2017; Primi et al., 2020), we hypothesized that these proxy scales would show good reliability despite their brevity. Next, we examined the validity of the ES-AMAS-P and the ES-AMAS-T by investigating the relationships between proxy and self-report scores. We expected positive associations across informants, reflecting some degree of convergence, while recognizing that parent, teacher, and child reports are not expected to be interchangeable. Rather, each informant may provide partially distinct information on children’s math anxiety, reflecting differences in the situations they can observe, their perspectives, and the manifestations of anxiety that may be accessible to them. Thus, we expected the multi-informant assessment to capture complementary aspects of the construct that may not be fully represented by any single informant. Furthermore, drawing on evidence linking math anxiety to poorer academic outcomes (e.g., Putwain & Wood, 2023; Wu et al., 2012), we hypothesized that higher levels of proxy-reported math anxiety would be associated with lower mathematical proficiency, which was assessed through both teachers’ evaluations and a standardized measure of mathematical competence.

As a fundamental step in evaluating the adequacy of a multi-informant approach, we also examined the explanatory power of parent-, teacher-, and self-reported math anxiety in relation to children’s mathematical competence. The primary objective was to investigate the relationships between these three perspectives (i.e., the parent- and teacher-proxy versions of the ES-AMAS and the self-report version administered to children) and children’s mathematical competence, while also disentangling the relative contribution of each informant. Specifically, we hypothesized that higher levels of children’s math anxiety, as reported by parents, teachers, and children themselves, would be associated with lower mathematical proficiency. Given that prior research has emphasized the potential impact of adults’ psychological characteristics on their perceptions of children’s functioning, we accounted for the potential effect of parents’ and teachers’ own math anxiety on their evaluations of children’s math anxiety by including these variables as covariates. This methodological choice was informed by evidence showing that multi-informant assessments often yield discrepant reports (De Los Reyes & Kazdin, 2005), likely reflecting the inherently subjective nature of each informant’s perspective. For example, parents may either overestimate or underestimate their child’s anxiety based on their emotional states, expectations, or cognitive biases. Consistently, Bögels and Phares (2008) showed that parents with elevated anxiety are more likely to perceive anxiety symptoms in their children—a phenomenon referred to as “parental distortion” (Griest et al., 1980)—wherein parental emotions or expectations systematically bias their perception of the child’s behavior (Griest et al., 1980). Moreover, because parents may not directly observe the school-based situations assessed by the AMAS, their ratings may partly reflect their perceptions and interpretations of how their child experiences mathematics, rather than direct observations of the child’s responses to the situations described in the scale. Parents’ own math anxiety may therefore influence how they perceive and evaluate their child’s experiences with mathematics. Therefore, to address these potential sources of bias and obtain a more accurate estimate of the relationship between math anxiety and mathematical competence, we accounted for the effect of adults’ math anxiety on their evaluations of children’s math anxiety. A covariance among informants’ ratings was also included, ensuring a more precise examination of the interplay among these variables.

## 2. Materials and Methods

### 2.1. Development of the Parent- and Teacher-Proxy Versions of the ES-AMAS

The parent- and teacher-proxy versions of the elementary school AMAS were designed to parallel the contents of the children’s self-report version, while adapting the instructions and wording to capture an external observer’s perspective. The original prompt for children (*“Please indicate how anxious you feel when doing these activities”*) was reformulated for parents (*“Please indicate how much anxiety your child shows in the described situations”*) and for teachers (*“Please indicate how much anxiety each student shows in the described situations”*). Item wording was similarly modified to suit the external observer’s perspective. For example, the self-report item *“When your teacher explains a new math topic”* was reformulated as *“When the teacher explains a new math topic”* (parent-proxy version) and *“When I explain a new math topic”* (teacher-proxy version). Adults are asked to evaluate the level of anxiety the child experiences when performing specific math activities on a scale from 1 (Little anxiety) to 5 (A lot of anxiety), basing their ratings on observable behavioral manifestations of the child’s anxiety and on information available to them through the child’s reports of their everyday experiences at home and at school. For each version, two age-specific forms were developed: One for parents and teachers of children aged 6–7 years and one for parents and teachers of children aged 8–11 years. These forms differed slightly in item content to align with the corresponding age-specific self-report versions (Caviola et al., 2017; Primi et al., 2020). Indeed, each self-report measure addresses slightly different situations, accounting for developmental differences in children’s experiences and school tasks. For example, in the self-report versions, the original item *“Listening to another student explain a math formula”* was adjusted to *“When another student solves a sum on the board”* for younger children (6–7 years; Primi et al., 2020) and *“Watching another student solve a math problem”* for older children (8–11 years; Caviola et al., 2017). This tailored approach ensured that the content of each version aligns with the developmental stages of the children being evaluated while maintaining conceptual equivalence across informants. A focus group involving parents and teachers was conducted to evaluate the clarity and comprehensibility of the scale: participants were asked to read the items and provide feedback on their wording and interpretability. Based on this feedback, both the items and the instructions were further refined.

### 2.2. Participants

A total of 1146 children (50.66% female; *M_age_* = 8.80, *SD* = 1.08) from three primary schools in central Italy participated in the study. Specifically, 14.49% of the participants were in the 1st grade, 17.71% in the 2nd grade, 21.99% in the 3rd grade, 19.81% in the 4th grade, and 26% in the 5th grade. Additionally, 869 of their parents (54.8% female; *M_age_* = 43.7, *SD* = 5.90) and 52 mathematics teachers (90.3% female; *M_age_* = 47.55, *SD* = 9.89) took part in the study. Each teacher provided ratings for multiple children, with the number of children rated by each teacher ranging from 3 to 26. For 38% of teachers, the number of children rated was fewer than 10. To recruit participants, the project was presented to a large selection of public primary schools in the Tuscany region. Three schools, located in two different provinces and situated in both urban and suburban areas, agreed to participate. Their demographic and educational characteristics can be considered representative of the wider regional population.

The participating schools adhere to the National Guidelines for the Mathematics Curriculum in Primary Schools. Regarding the development of arithmetic skills, students progressively develop their ability to perform basic arithmetic operations (addition, subtraction, multiplication, and division) with natural numbers, decimals, and integers. In the early grades, the focus is on mental calculation and simple operations, while more complex calculations, including those with decimals, are introduced in later grades. The curriculum aims to cultivate a deep understanding of mathematical operations and their practical applications while also promoting the use of fractions and the resolution of mathematical problems through computation.

The study’s aims and methodology were approved by the collegiate bodies of each participating school. All procedures adhered to the ethical guidelines established by the Italian Association of Psychology (2015, revised 2022), which align with the Declaration of Helsinki (1964/2013). Ethical approval was obtained from the Institutional Review Board of the University of Florence (Approval No. 341, 18 June 2024). Written informed consent was obtained from all participants before their inclusion in the study. Regarding the children, consent was obtained from both parents. Confidentiality and anonymity were strictly maintained throughout data collection and analysis.

### 2.3. Measures and Procedure

#### 2.3.1. Children’s Measures

The children completed the self-report version of the Abbreviated Math Anxiety Scale (Elementary School-Abbreviated Math Anxiety Scale-Self-report [ES-AMAS-SR]; Caviola et al., 2017; Primi et al., 2020), a nine-item self-report measure designed to assess math anxiety in primary school children. Consistent with the original version (Hopko et al., 2003), it assesses two dimensions of math anxiety: Learning-related math anxiety (5 items, e.g., *When another student solves a sum on the board/Watching another student solve a math problem*) and Evaluation-related math anxiety (4 items, e.g., *When you are taking a math test at school/Doing a written math test*). The scale has demonstrated good reliability and validity in Italian children (Caviola et al., 2017; Primi et al., 2020). Two age-specific forms of this scale are available, with item content adapted to reflect the experiences of children aged 6–7 and 8–11 years. In the present study, a pictorial response scale was employed to facilitate accurate self-reporting. Participants rated their anxiety in different math-related situations using a five-point pictorial scale, represented by boxes with progressively increasing fill, ranging from 1 (Little anxiety) to 5 (A lot of anxiety). This visual aid was designed to enable children to intuitively identify and select the level of anxiety experienced in each situation. This approach is consistent with literature suggesting that pictorial formats reduce the cognitive load required for abstract numerical translation, making the task more developmentally appropriate for young respondents (Borgers et al., 2000; Mellor & Moore, 2014). By lowering the cognitive demand of the assessment, such formats help ensure that children’s responses more accurately reflect their actual emotional state rather than their ability to navigate complex response structures (Mellor & Moore, 2014). Consequently, visually guided formats are generally more accessible and engaging for young respondents, thereby enhancing the overall accuracy and reliability of self-reports (Mahakwe et al., 2021).

Mathematical competence was assessed using the Written Operations subtest of the Calculation Skills Assessment Test 6–11 years—MT Group (AC-MT 6–11 years; Cornoldi et al., 2012), a standardized tool that included a range of collective (e.g., Written Operations, Number Judgment, Number Ordering) and individual subtests (e.g., Mental Calculation, Enumeration, Number Dictation) designed to assess mathematical abilities in Italian primary school children. The basic mathematical operations subtest was chosen for its alignment with fundamental mathematical competencies and their relevance to the Italian primary school curriculum. This subtest assesses children’s ability to perform basic arithmetic operations, focusing on addition and subtraction in the first and second grades, and all four basic operations in grades three to five. It consists of four items for first and second graders, and eight items for students in grades three to five. The difficulty level increases progressively, starting with simple single-digit calculations and advancing to more complex operations. This subtest measures children’s competence in performing fundamental mathematical calculations and understanding basic arithmetic principles. The task was administered collectively and concluded once 90% of children had completed the task. The score was determined by counting the number of correct answers. The raw score was then adjusted by dividing by the total number of items to yield a weighted score, ensuring comparability across grades.

The study protocol was administered to the children collectively during regular school hours. A trained examiner introduced the session with a brief explanation of anxiety and its manifestations and explained the response scale. Children were assured of the anonymity of their responses and encouraged to respond honestly. Given their still-developing reading skills, for first- and second-grade children, the examiner read each item aloud, pausing to allow all children to respond before proceeding. Third-, fourth-, and fifth-grade students completed the protocol independently after the examiner’s introduction. The entire procedure took approximately 40 min.

#### 2.3.2. Parents’ and Teachers’ Measures

The parent and teacher protocols included, alongside the proxy versions of the instrument assessing children’s math anxiety, a measure assessing the math anxiety of the adult raters themselves. Teachers were also asked to evaluate each student’s mathematical proficiency using a three-point scale, ranging from 1 (Below the expected level for his/her age) to 3 (Above the expected level for his/her age).

The ES-AMAS-P and the ES-AMAS-T were designed to parallel the contents of the self-report version of the scale (ES-AMAS-SR; Caviola et al., 2017; Primi et al., 2020). The instructions and the item formulation were modified to fit the perspective of external observers. Adults were asked to rate the level of anxiety the child experiences when performing specific math activities on a scale from 1 (Little anxiety) to 5 (A lot of anxiety), basing their ratings on observable behavioral manifestations of the child’s anxiety and on information available to them through the child’s reports of their everyday experiences at home and at school, including the child’s descriptions of and emotional responses to experiences involving mathematics. Consistent with the self-report version, separate forms were developed for parents and teachers of children aged 6 to 7 years and for those of children aged 8 to 11 years, with some item content adjusted accordingly.

The Abbreviated Math Anxiety Scale (AMAS; Hopko et al., 2003; Italian version, Primi et al., 2014) is a self-report instrument designed to assess math anxiety. It consists of nine items measuring two dimensions: Learning-related math anxiety (5 items; e.g., *Watching the teacher solve an equation on the board*) and Evaluation-related math anxiety (4 items; e.g., *Taking a math test*). Participants were asked to rate the extent of anxiety they have experienced in their studies in various math-related situations on a 5-point Likert scale ranging from 1 (Little anxiety) to 5 (A lot of anxiety).

Parents completed the protocols at home, with each protocol accompanied by detailed instructions. In this study, the ES-AMAS-P was administered to one parent per child, following prior evidence of dyadic invariance between mothers and fathers (Di Leonardo et al., 2023). Teachers completed the protocols in the classroom under the supervision of the examiner.

### 2.4. Statistical Analysis

An initial examination of missing data was conducted for the ES-AMAS and the AMAS. Cases with more than 10% missing responses were excluded from the analyses. Since the ES-AMAS consists of only nine items, participants were excluded if they had more than one missing response. Based on this criterion, 14 mothers, 17 fathers, one teacher, and three children were excluded because of missing responses on the respective ES-AMAS version. In addition, 7 mothers, 10 fathers, and one teacher were excluded because of missing responses on the AMAS. For the remaining missing values, the participant’s mean score on the corresponding subscale was used for imputation, in accordance with the established scoring procedure for the measure.

Subsequently, item distributions and descriptive statistics were examined to assess normality. A Confirmatory Factor Analysis (CFA) was then performed using the maximum likelihood method (ML) to evaluate the fit of the two-factor model. Model fit was assessed using the Comparative Fit Index (CFI; Bentler, 1990), the Tucker–Lewis Index (TLI; Tucker & Lewis, 1973), and the Root Mean Square Error of Approximation (RMSEA; Steiger, 1990). Following established guidelines, CFI and TLI values above 0.90 indicate acceptable fit, with values above 0.95 reflecting excellent fit, while RMSEA values below 0.08 indicate acceptable fit, and values below 0.05 indicate excellent fit (Hu & Bentler, 1999). Regarding the teacher-report data, given their nested structure, a multilevel CFA was initially considered. The ICC was 0.252 (95% CI [0.157, 0.349]), indicating that approximately 25% of the variance in teacher-reported math anxiety was attributable to between-teacher differences. However, despite attempts to estimate the multilevel model, the model failed to converge because of computational difficulties. Therefore, the factorial structure of the ES-AMAS-T was examined using a single-level CFA.

The reliability of the ES-AMAS was evaluated in terms of internal consistency using McDonald’s ω. Regarding validity, Pearson correlations were computed among the three versions of the ES-AMAS. Furthermore, Pearson or Spearman correlations were computed among ES-AMAS-SR, ES-AMAS-P, and ES-AMAS-T scores, children’s mathematical competence, and teacher-reported mathematics proficiency.

To examine the relationships between math anxiety reported by different informants (parents, teachers, and children) and children’s mathematical competence, while accounting for the potential effect of adults’ own math anxiety on their evaluations, a path analysis was conducted using R with the lavaan package (Rosseel, 2012). In this model, the Written Operations score served as the dependent variable, and the total scores of the ES-AMAS-P, ES-AMAS-T, and ES-AMAS-SR were entered as independent variables. Parents’ and teachers’ AMAS scores were included as covariates to account for the potential effect of adults’ math anxiety on their ratings of the children’s math anxiety. Because not all measures were available for all participants, the path analysis was conducted on the subset of participants for whom complete data were available for all variables included in the model. Accordingly, cases with missing data on any of the model variables were excluded using listwise deletion.

## 3. Results

### 3.1. Dimensionality

The analysis of item distributions (Table 1) showed that, for children, skewness indices ranged from −0.10 to 1.46, and kurtosis values from −1.32 to 1.07. Among parents, skewness ranged from 0.22 to 1.00, and kurtosis was between −0.90 and 0.55. For teachers, skewness was between 0.20 and 1.41, with kurtosis from −0.73 to 1.04. Minor deviations from normality were considered negligible (Ghasemi & Zahediasl, 2012).

**Figure 1:**
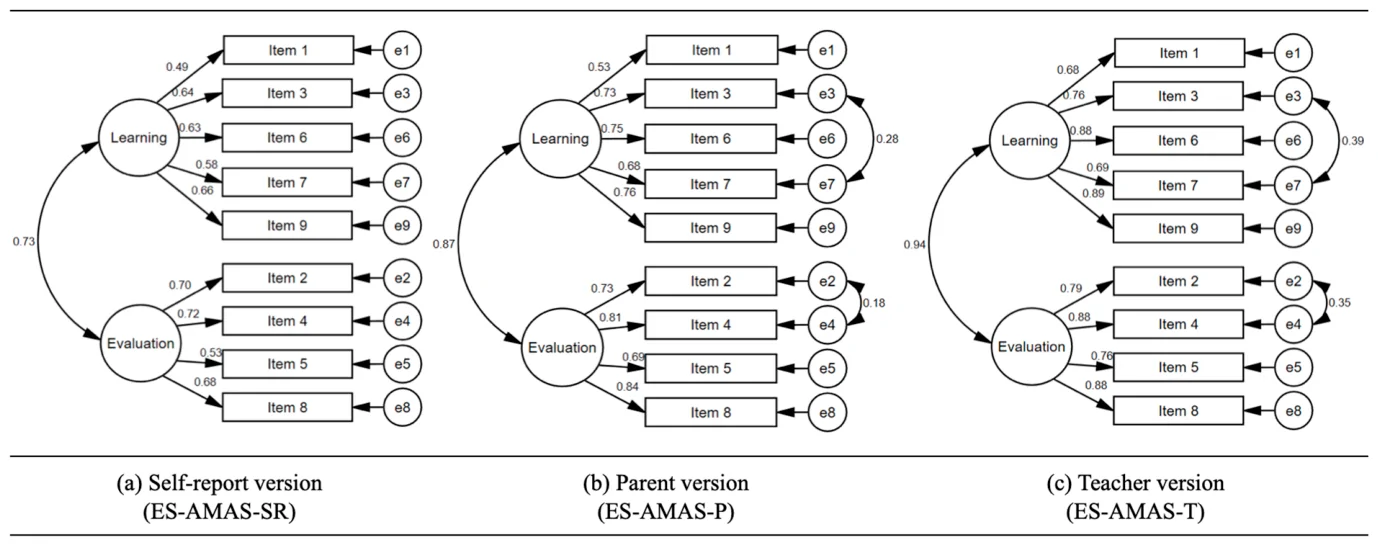
Two-factor structure of the three versions of the Elementary School—Abbreviated Math Anxiety Scale (ES-AMAS).

Consistent with previous research, the original two-factor structure was confirmed for the adapted ES-AMAS-SR incorporating the pictorial response scale. The model demonstrated acceptable fit indices (CFI = 0.941; TLI = 0.918; RMSEA = 0.073, 90% CI [0.063, 0.083]). Factor loadings ranged from 0.49 to 0.72, all exceeding 0.45 (*p* < 0.001), with a factor correlation of 0.73 (Figure 1a).

Results in the parents’ sample showed that the overall fit of the original two-factor model was not entirely adequate, since RMSEA was slightly greater than 0.08 (CFI = 0.950; TLI = 0.931; RMSEA = 0.093, 90% CI [0.082, 0.105]). Modification indices (MI) suggested adding error covariances between item 2 and item 4, as well as between item 6 and item 9. These modifications were theoretically justified by the content of the items: items 2 and 4 both refer to situations involving thinking about or taking a math test, whereas items 6 and 9 both refer to dealing with a new math topic. The modified model showed an adequate fit (CFI = 0.963, TLI = 0.945, RMSEA = 0.083, 90% CI [0.072, 0.096]). All factor loadings were significant and exceeded 0.50, ranging from 0.53 to 0.84 (*p* < 0.001). The correlation between the two factors was 0.83 (Figure 1b).

Similarly, in the teachers’ sample, the overall fit of the original two-factor model was not entirely adequate (CFI = 0.932; TLI = 0.906; RMSEA = 0.137, 90% CI [0.124, 0.151]). The modification indices (MI) suggested adding an error covariance between item 2 and item 4, which is theoretically justified because both items refer to becoming aware of or taking a written math test, and between item 3 and item 7, also theoretically justified, as both items refer to observing the execution of a math operation on the board. The modified model showed improved fit, although RMSEA remained above 0.08 (CFI = 0.961; TLI = 0.941; RMSEA = 0.108, 90% CI [0.095, 0.123]). All factor loadings were significant, ranging from 0.68 to 0.90, all exceeding 0.60 (*p* < 0.001). The correlation between the two factors was 0.94 (Figure 1c).

### 3.2. Reliability and Validity

Across all three versions, McDonald’s ω values for both the overall scale and the two subscales were good or adequate according to the EFPA guidelines (Evers et al., 2013). Furthermore, no improvement in omega values was observed after item elimination (Table 2).

The correlation matrix showed that both the total scores and subscale scores of the ES-AMAS-P and ES-AMAS-T were significantly and positively correlated with the corresponding scores of the ES-AMAS-SR. Additionally, significant positive correlations were observed between the total and subscale scores of the ES-AMAS-P and ES-AMAS-T. However, the small-to-medium magnitude of these correlations suggests that they capture related but unique aspects of children’s math anxiety (Table 3).

Regarding correlations among the ES-AMAS-SR, the ES-AMAS-P, and the ES-AMAS-T scores, children’s mathematical competence, and the mathematical proficiency ratings provided by teachers, the total score and subscale scores of the ES-AMAS-P, the ES-AMAS-T, and the ES-AMAS-SR showed significant negative correlations with both children’s mathematical competence and the teacher-reported mathematics assessment (Table 4; see Supplementary Table S1 for the full set of correlations). Both the nested structure of the teacher-report data and potential shared-rater effects, given that teachers provided ratings of both children’s math anxiety and mathematical competence, should be considered when interpreting the stronger association between teacher-reported math anxiety and mathematical competence.

**Figure 2:**
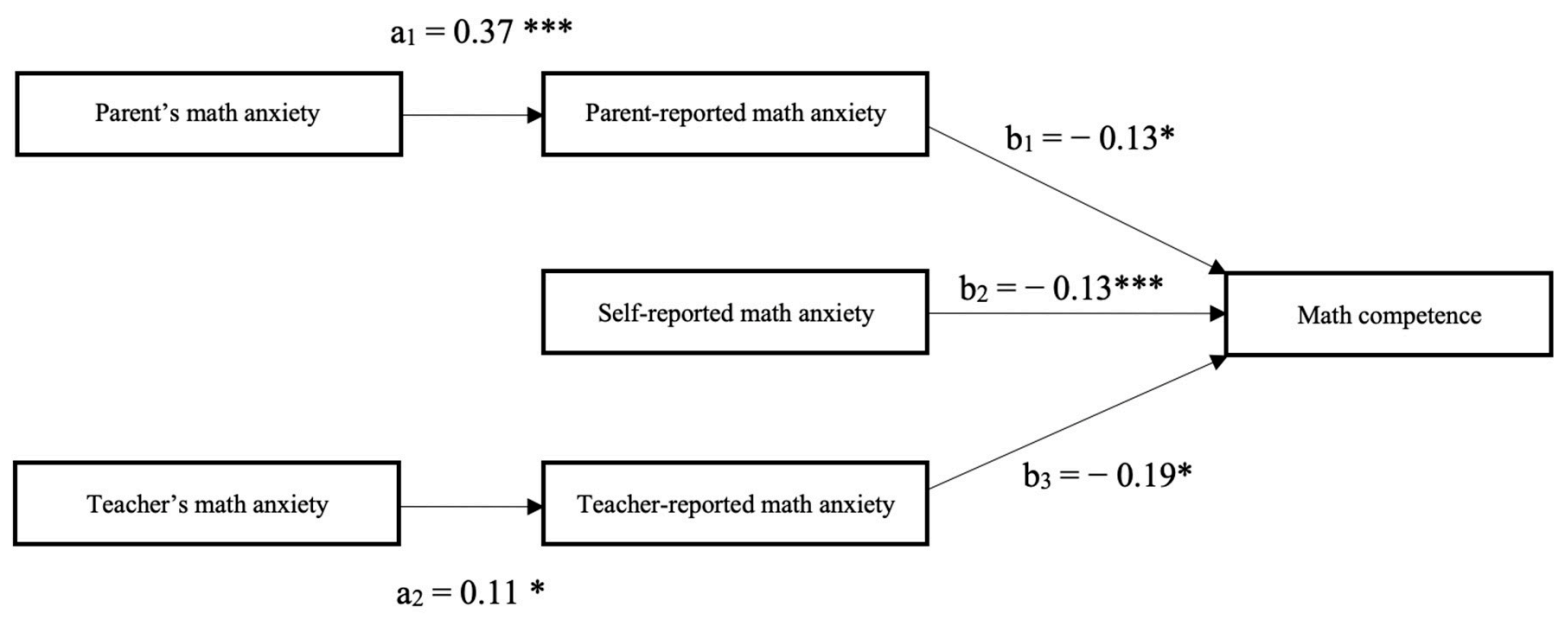
Path coefficients derived from the path model examining relationships between children’s math anxiety reported by different informants and children’s mathematical competence. Note. Coefficients (a and b) represent standardized estimates. * *p* < 0.05; *** *p* < 0.001.

Then, we tested a path model examining the association between children’s math anxiety, assessed from multiple informants (parents, teachers, and self-reports), and children’s mathematical competence, while also accounting for parents’ and teachers’ math anxiety as covariates (Figure 2). The path model was based on 370 complete observations, including data on all three assessments of children’s math anxiety (parent-, teacher-, and self-reported) as well as parents’ and teachers’ own math anxiety. The overall model showed an acceptable fit to the data, *χ*^2^(6) = 21.94, *p* = 0.001, CFI = 0.922, RMSEA = 0.085, 90% CI [0.049, 0.124]. Results indicated that all three perspectives on children’s math anxiety—parent-reported, teacher-reported, and self-reported—showed significant negative effects on children’s mathematical competence. Specifically, parent-reported math anxiety (*β* = −0.13, *p* < 0.05), teacher-reported math anxiety (*β* = −0.19, *p* < 0.001), and child self-reported math anxiety (*β* = −0.13, *p* < 0.05) each independently contributed to lower mathematical competence. The combined total effect of math anxiety from these three sources was significant and negative (*β*_total_ = −0.45, *p* < 0.001). Importantly, parents’ and teachers’ AMAS scores played a significant role in their respective perceptions of children’s math anxiety. Specifically, parental math anxiety showed a significant effect on parent-reported perceptions of children’s math anxiety (*β* = 0.37, *p* < 0.001), whereas teacher math anxiety had a smaller but still significant effect on teacher-reported perceptions of children’s math anxiety (*β* = 0.11, *p* < 0.05). However, the associations between parent- and teacher-reported math anxiety and children’s mathematical competence remained significant when accounting for these covariates.

## 4. Discussion and Conclusions

This study aimed to address the limitations of self-report tools in assessing psychological constructs in children by developing and validating a multi-informant battery for measuring math anxiety in children aged 6 to 11 years. First, after preliminarily confirming that the two-factor structure of the ES-AMAS-SR (Caviola et al., 2017; Primi et al., 2020) was retained following the adaptation of the response scale into a pictorial format for older children (third to fifth grade), the same structure was evaluated in the proxy versions (parent and teacher). The model demonstrated an adequate fit in the parent sample. A comparable pattern emerged in the teacher sample, where CFI and TLI values fell within acceptable ranges, supporting the adequacy of the hypothesized factor structure. The higher residual variance observed in the teacher ratings is likely attributable to the characteristics of the measurement context. Teachers provided evaluations for multiple children, introducing a dependency structure in the data that is not accounted for in single-level CFA and may inflate residual-based indices such as the RMSEA (e.g., Geldhof et al., 2014; Stapleton, 2006). Within this framework, standard single-level CFA may yield biased fit indices (Ulitzsch et al., 2017); therefore, the elevated RMSEA is more plausibly interpreted as reflecting measurement-related noise inherent to the multi-informant, multi-target assessment context, rather than a substantive misspecification of the model (Shi et al., 2019).

Moreover, the two multi-informant versions were positively correlated with the self-report measure of children’s math anxiety. However, the correlations among the different informants ranged from small to medium in magnitude, indicating partial rather than strong cross-informant agreement. This pattern is consistent with prior research showing that agreement between informants tends to be modest and may vary substantially depending on the construct being assessed (De Los Reyes & Kazdin, 2005). In particular, cross-informant agreement tends to be higher for more observable problems, such as externalizing problems, than for internalizing symptoms and subjective experiences, which are less directly observable (Achenbach et al., 1987). Similarly, research comparing child self- and parent proxy-reports of health-related quality of life has shown greater agreement for more concrete and observable domains, such as physical health, than for psychosocial domains (Galloway & Newman, 2017; Khanna et al., 2022). This distinction is particularly relevant to anxiety, as external observers may identify behavioral or emotional manifestations associated with anxiety without having direct access to the child’s subjective experience of anxious feelings. Moreover, given the internalizing nature of anxiety, adults’ ratings may reflect not only their perceptions of their children’s anxiety but also, to some extent, their own experiences. For example, Lyneham et al. (2008) reported limited cross-informant agreement in the assessment of school anxiety, with weak but significant associations between mother- and teacher-reported anxiety and no significant associations between child- and teacher-reported anxiety. Thus, the small-to-medium associations observed in the present study are broadly consistent with previous evidence on cross-informant agreement in the assessment of anxiety.

A further consideration concerns the nature of parents’ reports. Parents cannot directly observe all the situations described in the scale, particularly those occurring in the classroom. Therefore, when rating situations that they cannot directly witness, their responses may partly reflect inferences based on information provided by the child and on observable behavioral or emotional manifestations of anxiety, rather than direct observation of the specific situation. However, in a multi-informant assessment, parent reports are not intended to provide an objective measure of the child’s anxiety, but rather to capture the parent’s subjective perception of how the child experiences and responds to mathematics. This subjective perspective is an inherent component of multi-informant assessment and may provide complementary information to children’s self-reports and teachers’ reports. In particular, children’s reports of their everyday experiences at home and at school, together with observable behavioral or emotional manifestations of anxiety, may provide parents with relevant information about how children experience and respond to mathematics-related situations. Thus, the subjective nature of parent reports should be considered when interpreting their ratings, while recognizing that it also represents an important component of the multi-informant approach.

Nevertheless, the significant associations among the three versions suggest convergence between informants, while the small-to-medium magnitude of these associations also suggests that the perspectives provided by different informants may capture partially distinct aspects of children’s math anxiety. Consistent with this interpretation, research on cross-informant discrepancies has emphasized that discrepancies between informants should not necessarily be interpreted as measurement error, as different informants may provide partially different perspectives on the same phenomenon (Korelitz & Garber, 2016). Taken together, these findings highlight the added value of combining multiple sources to achieve a more nuanced and comprehensive understanding of children’s math anxiety. Future research should investigate the mechanisms underlying differences across informants and the factors that may account for these distinct perspectives.

Importantly, although adult math anxiety played a role in shaping their evaluations of children’s math anxiety, the proxy versions of the ES-AMAS captured children’s math anxiety independently of the adults’ own anxiety levels. Moreover, parents’ or teachers’ math anxiety did not confound the relationship between adults’ ratings of children’s math anxiety and children’s mathematical competence. In other words, accounting for adults’ own math anxiety helped clarify the unique contribution of their evaluations, supporting the use of the proxy instruments even when adult informants experience math-related anxiety, which is particularly relevant for assessments conducted in everyday educational settings.

This study further examined the relationship between math anxiety, as reported by parents, teachers, and children, and children’s mathematical competence. Scores from all informants showed negative correlations with children’s mathematics proficiency, as measured both by a standardized tool for assessing mathematical competence and teacher-reported evaluations. These findings suggested that perceptions of math anxiety, irrespective of the informant, were negatively associated with children’s mathematical competence. Moreover, parent-, teacher-, and self-reported math anxiety each independently had an effect on lower proficiency, with teachers’ evaluations having the most substantial impact. This indicated that math anxiety, as assessed by multiple informants, collectively and uniquely contributed to explaining differences in mathematical outcomes.

Overall, these results support the value of a multi-informant approach to the assessment of children’s math anxiety and highlight the importance of integrating information from different sources to better understand the complex relationship between math anxiety and children’s mathematical competence. The small-to-medium inter-informant correlations, together with the unique associations of each informant’s report with children’s mathematical competence, suggest that the three versions capture partially overlapping but distinct aspects of children’s math anxiety. The three sources may therefore provide complementary information, offering a more nuanced and comprehensive assessment of this construct.

The results also underscore the need for targeted interventions addressing both mathematical skills and the emotional dimensions of learning mathematics. Gamification, which integrates game elements into learning, appears promising for increasing engagement and reducing math anxiety. Recent studies (e.g., Balt et al., 2022) suggest that the use of math games and training software can significantly enhance students’ perceived competence while alleviating math anxiety. Gamification provides a more enjoyable and accessible experience with mathematics, helping students shift their perceptions of the subject from intimidating to engaging. Moreover, structuring games to include progressively more challenging tasks that match students’ skill levels can help disrupt the cycle of avoidance and anxiety and support more confident engagement with mathematics (Levine & Pantoja, 2021).

Despite these contributions, the study has several limitations. First, regarding the teacher-report data, the factorial structure was examined using a single-level CFA approach. This methodological choice was primarily driven by computational difficulties encountered when attempting to estimate a multilevel CFA to account for the nested structure of the data. Specifically, despite attempts to estimate the multilevel model, a stable solution could not be obtained in our sample because of convergence difficulties (Brown, 2015; Hox et al., 2017). However, the single-level approach does not explicitly account for the non-independence of observations arising from the fact that multiple children within the same class were rated by the same teacher and shared the same assessment context. This unmodeled dependency may have affected the residual variance and, consequently, the fit of the teacher-report model, potentially contributing to the higher RMSEA observed in comparison with the other models. Nevertheless, the extent to which the higher RMSEA reflects the unmodeled clustering, model misspecification, or other sources of variability cannot be determined from the present analyses. Similarly, the relatively stronger association between teacher-reported math anxiety and children’s mathematical competence should be interpreted with caution, given the non-independence of teacher ratings. However, the stronger association specifically observed with teachers’ evaluation of students’ mathematical proficiency may also be partly attributable to shared-informant effects, as both children’s math anxiety and their mathematical proficiency were assessed by the same teacher. The same consideration regarding the nested structure applies to the SEM analyses, in which teachers’ reports were likewise nested within classes, but this dependency was not explicitly modeled. At the same time, it is important to recognize that having teachers rate multiple children is an inherent feature of teacher-report data in multi-informant research, as teachers typically provide information on several students within the same classroom. Therefore, the fact that teachers provided ratings for multiple children should not, in itself, be regarded as a methodological limitation, but as a characteristic of teacher-report data that needs to be appropriately accounted for in the statistical analyses. Future studies should include samples with a sufficiently large and appropriately structured number of clusters and observations to allow the nested structure of teacher-report data to be explicitly modeled using multilevel CFA and SEM. Such analyses could help clarify whether and to what extent accounting for the nested structure of teacher-report data affects parameter estimates, associations with mathematical competence, and model fit.

A further limitation concerns the incomplete availability of multi-informant data. Because of the multi-informant design, data from parents and teachers were available only for a subset of the children, as some parents or teachers did not provide consent to participate or did not return the completed questionnaires. Consequently, corresponding parent- or teacher-proxy assessments were not available for all children. This differential availability of informant data resulted in a reduced analytic sample for the structural equation modeling analyses. In these analyses, cases with missing data on any of the variables included in the respective model were excluded using listwise deletion. The resulting reduction in sample size may have limited statistical power and increased uncertainty around the parameter estimates. Moreover, because the availability of parent and teacher data may not have been independent of characteristics of the children or their families, systematic differences between children with and without complete multi-informant data cannot be excluded. Therefore, the possibility of selection bias should be acknowledged. Future studies should seek to maximize participation across informants and assess potential differences between participants with complete versus incomplete multi-informant data.

Moreover, although the effect of adult math anxiety on the evaluation of children’s math anxiety was controlled, other adult factors (e.g., parental involvement in math-related activities, parents’ educational background, or teacher expectations) may also shape their perception of children’s math anxiety (De Los Reyes & Kazdin, 2005). Future research should examine these potential effects more systematically.

Additionally, the study focused solely on the relationship between math anxiety and proficiency on basic math tasks. Therefore, the identified relationships may not fully capture the connection between math anxiety and a broader range of mathematical activities, such as problem-solving, reasoning, or applied mathematics. Future studies should also examine the relationship between the multi-informant ES-AMAS and other measures of math anxiety and related constructs to establish convergent validity.

Moreover, this study was correlational, and the temporal stability of the measures was not examined. As the findings do not establish a causal relationship between math anxiety and mathematical proficiency, longitudinal designs could be employed in future studies to monitor math anxiety over time, using age-appropriate versions of the AMAS to examine both developmental trajectories and potential causal pathways linking math anxiety to academic outcomes.

It is also important to acknowledge the limitations of self-report measures when used with young children. Although the study aimed to develop a multi-informant battery, relying on self-reported measures in this age group introduces the risk of response biases due to their developing cognitive and emotional capacities, which may affect the accuracy of their self-reports. While the multi-informant structure addresses this limitation, future studies could also integrate innovative approaches—such as gamified assessment tools—to better capture math anxiety in children aged 6 to 11.

Finally, collecting information from multiple informants is essential, as children’s behaviors and emotional states can vary significantly across contexts (Dirks et al., 2012). Combining information from parents, teachers, and the child, greater accuracy can be achieved in assessing anxiety disorders, especially when their manifestations are not consistently observable across different settings (Comer & Kendall, 2004). Although anxiety symptoms differ from those of other disorders requiring cross-setting assessment (e.g., ADHD), capturing contextual variations in math anxiety remains essential. However, math anxiety, as currently measured, is primarily tied to the school context. For instance, while the multi-informant ES-AMAS facilitates the collection of data from the distinct perspectives of children, parents, and teachers, all three ES-AMAS versions measure math anxiety exclusively in relation to the school context, which may limit the understanding of how math anxiety might manifest in everyday, extra-school contexts (e.g., handling money, playing games involving numbers, or solving numerical problems in daily life). Future research should develop and validate instruments that capture math anxiety across both school and non-school settings. Extending assessment to include math anxiety manifestations outside school would provide a more complete picture of the child’s experience and facilitate more effective, context-sensitive interventions (De Los Reyes et al., 2015; Essau et al., 2000; Hawley & Weisz, 2003).

Despite these limitations, the availability of a multi-informant battery for assessing math anxiety in children represents an important step forward. The brevity and psychometric strength of these tools make them suitable for both research and applied settings, enabling early identification of at-risk children, evaluation of intervention outcomes, and improved educational and emotional support for children struggling with math anxiety.

## Figures and Tables

**Table 1:** Means (*M*), standard deviations (*SDs*), skewness, and kurtosis of the nine items of three versions of the ES-AMAS.

	Self-Report Version (ES-AMAS-SR) (*n* = 1146 Ratings)				Parent Version (ES-AMAS-P) (*n* = 869 Ratings)				Teacher Version (ES-AMAS-T) (*n* = 621 Ratings)			
Item	*M*	*SD*	Skewness	Kurtosis	*M*	*SD*	Skewness	Kurtosis	*M*	*SD*	Skewness	Kurtosis
1	2.00	1.26	1.07	0.08	1.97	1.09	0.92	−0.04	2.19	1.19	0.66	−0.61
2	2.80	1.36	0.24	−1.10	2.33	1.12	0.53	−0.49	2.62	1.09	0.29	−0.55
3	1.87	1.25	1.27	0.40	1.87	0.93	0.97	0.55	1.86	0.95	0.83	−0.15
4	2.75	1.38	0.27	−1.14	2.61	1.13	0.36	−0.56	2.74	1.15	0.23	−0.73
5	2.82	1.45	0.17	−1.32	2.74	1.23	0.27	−0.90	2.42	1.14	0.48	−0.53
6	2.23	1.33	0.78	−0.56	2.06	1.02	0.71	−0.19	2.16	1.11	0.74	−0.21
7	1.76	1.17	1.46	1.07	1.71	0.85	1.00	0.34	1.51	0.74	1.32	1.04
8	3.14	1.42	−0.10	−1.28	2.80	1.14	0.22	−0.69	2.76	1.06	0.20	−0.53
9	2.43	1.39	0.58	−0.94	2.27	1.10	0.58	−0.43	2.37	1.17	0.55	−0.56

Note. ES-AMAS-P = Elementary School—Abbreviated Math Anxiety Scale—Parent-report; ES-AMAS-T = Elementary School—Abbreviated Math Anxiety Scale—Teacher-report; ES-AMAS-SR = Elementary School—Abbreviated Math Anxiety Scale—Self-report. Likert scale ranges from 1 = Little anxiety to 5 = A lot of anxiety.

**Table 2:** McDonald’s ω coefficients for the subscales and the total scores of the three versions of the ES-AMAS.

	Self-Report Version (ES-AMAS-SR)	Parent Version (ES-AMAS-P)	Teacher Version (ES-AMAS-T)
Learning subscale	0.74 (95% CI [0.72–0.76])	0.88 (95% CI [0.86–0.89])	0.89 (95% CI [0.88–0.91])
Evaluation subscale	0.75 (95% CI [0.73–0.77])	0.86 (95% CI [0.84–0.87])	0.90 (95% CI [0.89–0.92])
Total scale	0.82 (95% CI [0.81, 0.84])	0.90 (95% CI [0.89, 0.91])	0.94 (95% CI [0.93, 0.95])

Note. ES-AMAS = Elementary School—Abbreviated Math Anxiety Scale.

**Table 3:** Pearson’s correlations between the subscales and the total scores of the three versions of the ES-AMAS.

	Self-Report-Parent-Report	Self-Report-Teacher-Report	Parent-Report-Techer-Report
1. Learning subscale score	0.277 **	0.230 **	0.260 **
2. Evaluation subscale score	0.381 **	0.277 **	0.261 **
3. Total score	0.369 **	0.297 **	0.283 **

Note. ES-AMAS = Elementary School—Abbreviated Math Anxiety Scale. ** *p* < 0.01.

**Table 4:** Pearson’s or Spearman’s correlations between the total scores of the three versions of the ES-AMAS, mathematical competence, and teacher-provided mathematics assessment.

	Mathematical Competence (Pearson’s r)	Teacher’s Mathematics Assessment (Spearman’s ρ)
1. Self-reported math anxiety (ES-AMAS-SR)	−0.230 **	−0.314 **
2. Parent-reported math anxiety (ES-AMAS-P)	−0.225 **	−0.308 **
3. Teacher-reported math anxiety (ES-AMAS-T)	−0.324 **	−0.577 **

Note. ** *p* < 0.01.

## Data Availability

The data supporting the findings of this study are not openly available due to sensitivity concerns but can be obtained from the corresponding author upon request.

## Supplementary Materials

The following supporting information can be downloaded at: https://www.mdpi.com/article/10.3390/bs16101838/s1, Table S1: Pearson’s or Spearman’s correlations between the subscales and the total scores of the three versions of the ES-AMAS, mathematical competence, and teacher-provided mathematics assessment.
