# Supplementary material for: Towards a Multi-Informant Assessment of Math Anxiety in Primary School Children
Source: Behav Sci (Basel). 2026 Oct 8;16(10):1838. doi: 10.3390/bs16101838 (PMC13701305; doi:10.3390/bs16101838)
Supplement: Supplementary file 1 [file behavsci-16-01838-s001.zip › behavsci-4461539-supplementary.pdf]

Supplementary materials

Table S1. Pearson's or Spearman's correlations between the subscales and the total scores of the three versions of the ES-AMAS, mathematical competence, and teacher-provided mathematics assessment.

	1	2	3	4	5	6	7	8	9	10	11
1. Learning-P	-										
2. Evaluation-P	0.735 **	-									
3. ES-AMAS-P	0.931 **	0.932 **	-								
4. Learning-T	0.260 **	0.262 **	0.283 **	-							
5. Evaluation-T	0.218 **	0.261 **	0.260 **	0.847 **	-						
6. ES-AMAS-T	0.249 **	0.272 **	0.283 **	0.965 **	0.957 **	-					
7. Learning-SR	0.277 **	0.252 **	0.284 **	0.230 **	0.222 **	0.235 **	-				
8. Evaluation-SR	0.313 **	0.381 **	0.373 **	0.282 **	0.277 **	0.291 **	0.576 **	-			
9. ES-AMAS-SR	0.332 **	0.355 **	0.369 **	0.289 **	0.282 **	0.297 **	0.892 **	0.883 **	-		
10. Mathematical competence	−0.197 **	−0.223 **	−0.225 **	−0.320 **	−0.301 **	−0.324 **	−0.227 **	−0.181 **	−0.230 **	-	
11. Mathematics assessment	−0.269 **	−0.294 **	−0.308 **	−0.549 **	−0.558 **	−0.577 **	−0.277 **	−0.269 **	−.314 **	0.414 **	-

Note. ES-AMAS-P = Elementary School - Abbreviated Math Anxiety Scale – Parent-report, ES-AMAS-T = Elementary School - Abbreviated Math Anxiety Scale – Teacher-report, ES-AMAS-SR = Elementary School - Abbreviated Math Anxiety Scale – Self-report. ** $p < .01$.
